# Two complete mitochondrial genomes of the barnacle *Lepas anatifera* Linnaeus, 1758 (Scalpellomorpha, Lepadidae) implying the possibility of cryptic speciation

**DOI:** 10.1080/23802359.2022.2086497

**Published:** 2022-06-20

**Authors:** Xiao-Nie Lin, Li-Sha Hu, Yun-Wei Dong

**Affiliations:** a The Key Laboratory of Mariculture, Ministry of Education, Fisheries College, Ocean University of China, Qingdao, PR China; b Function Laboratory for Marine Fisheries Science and Food Production Processes, Pilot National Laboratory for Marine Science and Technology, Qingdao, PR China

**Keywords:** Cryptic speciation, *Lepas*, mitochondrial genome, the northwest Pacific

## Abstract

The barnacle *Lepas anatifera* Linnaeus, 1758 (Scalpellomorpha, Lepadidae) is a worldwide distributed species. For investigating its genetic diversity in the northwest Pacific, two complete mitochondrial genomes were determined and analyzed. The lengths of the two complete mitogenomes were 15,708 bp and 15,703 bp, respectively. Both of them contained typical 37 genes with an identical order to *L. anserifera* Linnaeus, 1767 and *L. australis* Darwin, 1851 mitogenome. Except for *ND1* and *ND2*, 11 protein-coding genes (PCGs) started with an ATN initiation codon (ATA, ATG, ATC, and ATT). Twelve PCGs were terminated with TAA or TAG stop codon, whereas *ND1* possessed an incomplete termination codon (T–). Phylogenetic analysis revealed that *L. australis* and *L. anserifera* clustered together, and then with *L. anatifera*. The distinct genetic distances (0.17) based on concatenated sequence of 13 PCGs between the two mitogenomes of *L. anatifera* suggest the existence of cryptic speciation. Additional samples from multiple localities should be collected and analyzed to deepen the understanding of cryptic diversity within the northwest Pacific.

The goose barnacles of the genus *Lepas* have been found in the surface drifters of all oceans, and are considered ideal candidates to investigate genetic diversity and biogeographical patterns due to their cosmopolitan distribution (Schiffer and Herbig 2016). Mitochondrial DNA sequences have been successfully applied to identify intraspecies and interspecies genetic variations in phylogeographical studies (Baek et al. 2016). In the genus *Lepas*, complete mitogenomes of *L*. *anserifera* and *L*. *australis* have been reported. For the cosmopolitan *L. anatifera*, four regional subgroups (coastal Chile, Oregon, Indo-Pacific, Atlantic) and a global group were identified using *CO1* and *16S* rRNA (Schiffer and Herbig 2016). To get a better understanding of the genetic diversity of *L. anatifera* in the northwest Pacific, two specimens were collected from different biogeographic realms, the temperate northern Pacific realm, and the central Indo-Pacific realm (Spalding et al. 2007), and their complete mitogenomes were first sequenced in the present study.

One of the specimens (voucher number: LInE-Mcr001) was collected from a buoy in the Kuroshio Extension (KE) region (146.10°E, 32.00°N), the other one (LInE-Mcr002) was collected from the South China Sea (SCS) (119.00°E, 20.00°N), and following policies and procedures of the Ocean University of China bioethical committee. Two specimens were deposited at the Laboratory of Intertidal Ecophysiology, Fisheries College, Ocean University of China (Sha-Sha Cheng, chengshasha@ouc.edu.cn). Total genomic DNA was extracted using a modified cetyltrimethylammonium bromide (CTAB) method (Ding et al. 2018), and a 500-bp paired-end library was constructed for each specimen and sequenced using Illumina NovaSeq 6000 platform (BIOZERON Co., Ltd., Shanghai, China). Total of 6.4 Gb and 6.6 Gb of 150 bp paired-end length data, with 42,776,990 and 44,301,460 reads, were generated for the SCS and KE specimens, respectively. These reads were assembled separately for two mitogenomes using GetOrganelle v1.6.4 (Jin et al. 2020) with the mitogenome of *L*. *anserifera* as a reference (GenBank accession no. NC_026576.1). Annotations of tRNAs and rRNAs were generated in the MITOS web server (Bernt et al. 2013). Protein-coding genes (PCGs) were annotated based on the reference genome of *L. anserifera*. Bayesian’s inference (BI) phylogenetic tree was constructed using the plug-in MrBayes program in PhyloSuite v.1.2.1 (Zhang et al. 2020) based on nucleotide sequences of 13 PCGs and two rRNA genes of *L*. *anatifera* and other cirripeds using *Tetraclita japonica* as an outgroup. The best-fitting substitution model and partitioning schemes were selected using PartitionFinder v.2.1.1 (Lanfear et al. 2017). Genetic distances were calculated with the concatenated sequence of 13 PCGs based on the Kimura-2-parameter model using MEGA X (Kumar et al. 2018).

The lengths of complete mitogenomes of the KE and the SCS specimens were 15,708 bp (GenBank accession no. ON060685) and 15,703 bp (GenBank accession no. ON060686), respectively. Both of them owned 13 PCGs, two rRNA genes, 22 tRNAs genes, and two non-coding regions. The nucleotide composition of the two mitogenomes was similar, and the A + T contents of the KE specimen and SCS specimen were 66.76% and 67.52%, respectively. Except for *ND1* (start with GTG) and *ND2* (start with TTG), 11 PCGs started with an ATN (ATA, ATG, ATC, and ATT) initiation codon. Except *ND1* possessed an incomplete termination codon (T–), 12 PCGs were terminated with TAA or TAG stop codon. The two mitogenomes of *L. anatifera* shared the same gene order as mitogenomes of *L*. *anserifera* and *L*. *australis*. Except 10 tRNA genes (*trnF*, *trnH*, *trnP*, *trnI*, *trnY*, *trnC*, *trnL1*, *trnV*, *trnK*, and *trnQ*), four PCGs (*ND1*, *ND4*, *ND4L*, and *ND5*), and two rRNA genes (*rrnL* and *rrnS*), all rest genes were encoded on the heavy strand.

The phylogenetic tree revealed that *L. australis* and *L. anserifera* clustered together, and then with *L*. *anatifera* (Figure 1). The interspecific genetic distances between each two species pairs in the family Lepadidae ranged from 0.29 to 0.32, while the intraspecies genetic distance of *L. anatifera* in the present study was 0.17 (Figure 1). Although the intraspecific variation between the two *L. anatifera* individuals was less than the interspecific variation, it is undeniable that there was already a large genetic differentiation of the mitochondrial DNA between the two *L. anatifera* individuals, implying the existence of cryptic speciation of *L*. *anatifera* in the northwest Pacific. This result was consistent with the result as described by Schiffer and Herbig (2016) that cryptic speciation of *L*. *anatifera* existed in coastal Chile, Oregon, Indo-Pacific, and Atlantic. Additional samples from multiple localities should be collected and analyzed to deepen the understanding of the cryptic speciation of *L. anatifera* in the northwest Pacific.

**Figure 1.**
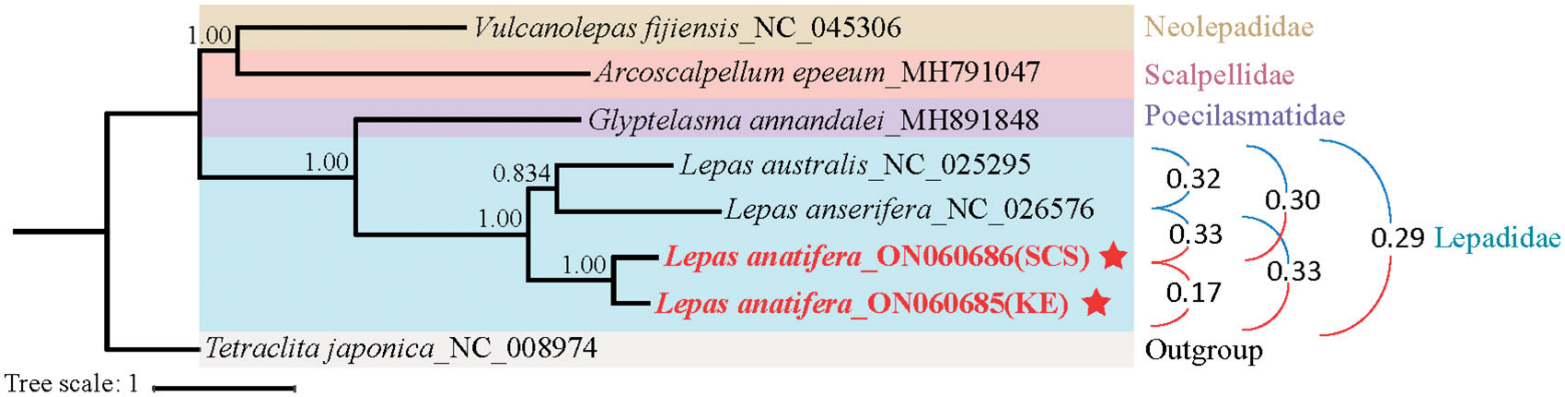
Bayesian’s inference (BI) phylogenetic tree for Scalpellomorpha order based on the nucleotide sequence data of 13 PCGs and two rRNA genes of *L*epas *anatifera* and other five species belonging to four related families of Scalpellomorpha order. Stars represent two complete mitogenomes first sequenced in the present study. The interspecific and intraspecies genetic distances were shown in the right of the figure.

## Data Availability

The genome sequence data that support the findings of this study are openly available in GenBank of NCBI at https://www.ncbi.nlm.nih.gov/ under the accession number ON060685 and ON060686. The associated BioProject, SRA, and BioSample numbers are PRJNA821487, SRR18537166–SRR18537167, and SAMN27103655–SAMN27103656, respectively.

## References

[CIT0001] Baek YS, Min GS, Kim S, Choi HG. 2016. Complete mitochondrial genome of the Antarctic barnacle *Lepas australis* (Crustacea, Maxillopoda, Cirripedia). Mitochondrial DNA A DNA Mapp Seq Anal. 27(3):1677–1678. 25228375 10.3109/19401736.2014.958724

[CIT0002] Bernt M, Donath A, Juhling F, Externbrink F, Florentz C, Fritzsch G, Putz J, Middendorf M, Stadler PF. 2013. MITOS: improved de novo metazoan mitochondrial genome annotation. Mol Phylogenet Evol. 69(2):313–319. 22982435 10.1016/j.ympev.2012.08.023

[CIT0003] Ding MW, Wang ZK, Dong YW. 2018. Food availability on the shore: linking epilithic and planktonic microalgae to the food ingested by two intertidal gastropods. Mar Environ Res. 136:71–77. 29478767 10.1016/j.marenvres.2018.02.005

[CIT0004] Jin JJ, Yu WB, Yang JB, Song Y, DePamphilis CW, Yi TS, Li DZ. 2020. GetOrganelle: a fast and versatile toolkit for accurate de novo assembly of organelle genomes. Genome Biol. 21(1):1–31. 10.1186/s13059-020-02154-5 PMC7488116 32912315

[CIT0005] Kumar S, Stecher G, Li M, Knyaz C, Tamura K. 2018. MEGA X: molecular evolutionary genetics analysis across computing platforms. Mol Biol Evol. 35(6):1547–1549. 29722887 10.1093/molbev/msy096 PMC5967553

[CIT0006] Lanfear R, Frandsen PB, Wright AM, Senfeld T, Calcott B. 2017. PartitionFinder 2: new methods for selecting partitioned models of evolution for molecular and morphological phylogenetic analyses. Mol Biol Evol. 34(3):772–773. 28013191 10.1093/molbev/msw260

[CIT0007] Schiffer PH, Herbig HG. 2016. Endorsing Darwin: global biogeography of the epipelagic goose barnacles *Lepas* spp. (Cirripedia, Lepadomorpha) proves cryptic speciation. Zool J Linn Soc. 177(3):507–525.

[CIT0008] Spalding MD, Fox HE, Allen GR, Davidson N, Ferdaña ZA, Finlayson M, Halpern BS, Jorge MA, Lombana A, Lourie SA, et al. 2007. Marine ecoregions of the world: a bioregionalization of coastal and shelf areas. BioScience. 57(7):573–583.

[CIT0009] Zhang D, Gao FL, Jakovlić I, Zou H, Zhang J, Li WX, Wang GT. 2020. PhyloSuite: an integrated and scalable desktop platform for streamlined molecular sequence data management and evolutionary phylogenetics studies. Mol Ecol Resour. 20(1):348–355. 31599058 10.1111/1755-0998.13096

